# Reproducibility and inter-observer agreement of Greulich-Pyle protocol to estimate skeletal age among female adolescent soccer players

**DOI:** 10.1186/s12887-020-02383-4

**Published:** 2020-10-26

**Authors:** Yuri V. Faustino-da-Silva, Diogo V. Martinho, Manuel J. Coelho-e-Silva, João Valente-dos-Santos, Jorge Conde, Tomás G. Oliveira, Enio R. V. Ronque, Ricardo R. Agostinete, Rômulo A. Fernandes, Lauren B. Sherar

**Affiliations:** 1 Scientific Research Group Related to Physical Activity (GICRAF), Laboratory of InVestigation in Exercise (LIVE), Department of Physical Education, Sao Paulo State University “Júlio de Mesquita Filho” (UNESP), Presidente Prudente,, São Paulo, Brazil; 2 University of Coimbra, FCDEF, Coimbra, Portugal; 3 University of Coimbra, CIDAF (uid/dtp/04213/2020), Coimbra, Portugal; 4 Faculty of Physical Education and Sport, Lusófona University, Lisbon, Portugal; 5 Department of Clinical Physiology, School of Health and Technology, Polytechnic Institute of Coimbra, Coimbra, Portugal; 6 Education Ministry, Lisbon, Portugal; 7 Londrina State University, Study and Research Group in Physical Activity and Exercise (GEPAFE), Londrina, Brazil; 8 School of Sport, Exercise and Health Sciences, Loughborough University, Loughborough, UK

**Keywords:** Youth sports, Female athlete, Biological maturation, Bone age, Atlas method

## Abstract

**Background:**

Skeletal age (SA) is considered the best method of assessing biological maturation. The aim of this study was to determine intra-observer (reproducibility) and inter-observer agreement of SA values obtained via the Greulich-Pyle (GP) method. In addition, the variation in calculated SAs by alternative GP protocols was examined.

**Methods:**

The sample was composed of 100 Portuguese female soccer players aged 12.0–16.7 years. SAs were determined using the GP method by two observers (OB1: experience < 100 exams using GP; OB2: experience > 2000 exams using several methods). The radiographs were examined using alternative GP protocols: (wholeGP) the plate was matched to the atlas as an overall approach; (30-boneGP) bone-by-bone inspections of 30-bones; (GPpmb) bone-by-bone inspections of the pre-mature bones only. For the 30-boneGP and GPpmb approaches, SA was calculated via the mean (M) and the median (Md).

**Results:**

Reproducibility ranged 82–100% and 88–100% for OB1 and OB2, respectively. Inter-observer agreement (100 participants multiplied by 30 bones) was 92.1%. For specific bones, agreement rates less than 90% were found for scaphoid (81%), medial phalange V (83%), trapezium (84%) and metacarpal V (87%). Differences in wholeGP SAs obtained by the two observers were moderate (d-cohen was 0.79). Mean differences between observers when using bone-by bone SAs were trivial (30-boneGP: d-cohen less than 0.05; GPpmb: d-cohen less than 0.10). The impact of using the mean or the median was negligible, particularly when analyses did not include bones scored as mature.

**Conclusion:**

The GP appeared to be a reasonably reproducible method to assess SA and inter-observer agreement was acceptable. There is evidence to support a recommendation of only scoring pre-mature bones during later adolescence. Further research is required to examine whether these findings are consistent in younger girls and in boys.

**Supplementary information:**

**Supplementary information** accompanies this paper at 10.1186/s12887-020-02383-4.

## Background

The study of growth status, biological maturation, and physical performance is central to sports sciences, human biology, and pediatrics. Growth refers to changes in body size and has implications for proportionality, shape, and composition [1]. Maturation is more difficult to define and refers to the progress toward the adult (mature) state. This occurs in all tissues and organs at different timing and rates, affecting functions and metabolism. Skeletal age (SA) refers to the degree of skeletal maturation and can be examined via a standardized radiographs (usually of the left hand and wrist). Although several indicators of biological maturation are available (e.g. secondary sex characteristics, age at peak velocity, predicted percentage of mature stature), SA is frequently considered the gold standard indicator of biological maturity, partly because it can be applied from fetal life through childhood and the second decade of life [1].

Assessment of biological maturation is common in youth sports. It has been recognized that maturity status impacts performance [2–5], injury [6–8], and selection [9]. Many studies have assessed the SA of adolescents participating in team sports (e.g. soccer, hockey) and found, in general, that players were early, maturing, taller, heavier and stronger [10, 11]. Malina et al. [12] suggested that one reason for this observation is that, among male adolescent soccer players late maturers tended to be systematically excluded during years of maximal growth while those classified as early or average maturing are selected and/or promoted by coaches and club administrators and this became even more evident as the players got older and specialized in one sport. Although the same rationale could be applied to female adolescent athletes, the evidence is scarce.

The Greulich-Pyle (GP) method is often used to obtain a SA estimate and involves comparing each individual bone against pictorial standards [13]. Across published literature, however, there are various versions of the GP protocol and often a lack of detail regarding the methods used to estimate SA. For example, one study [14] stated “*skeletal maturation was evaluated by the determination of bone age (BA) according to the GP method*” (page 626) and did not detail the GP procedures, specifically whether the radiograph was examined as a whole (wholeGP) or via bone by bone. A more recent study [15] described the biological maturity variation in body size, functional capacities, and sport-specific technical skills of 60 male Brazilian adolescent soccer players. The following was stated in the methodology: “*the left hand-wrist radiographs were obtained in a specialized laboratory and the GP method was adopted to estimate SA*” (page 465). The original authors prescribed that after identifying the standard which most closely resembles the film being assessed, one should proceed to make a more detailed comparison of the individual bones [13]. In practice, GP SAs seemed not to be properly obtained based on the SA of the standard plate which the film of a young person most closely matches, thus excluding variation among bones. Research is required to ascertain the error associated with different methods of examination to inform future studies.

The present study aimed to examine the intra-observer reproducibility and inter-examiner agreement using a variation in GP protocols: a) overall (wholeGP) or bone-by-bone approach; b) inspection of all bones (30-boneGP) or solely the pre-mature bones (GPpmb) c) calculating SA using the mean or the median values. It was hypothesized that agreement rates would be higher when observers follow a wholeGP and bone-by-bone approaches (30-boneGP; GPpmb).

The present study was aimed to examine the intra-observer reproducibility and inter-examiner agreement following concurrent GP protocols. Firstly, estimates obtained using an overall (wholeGP) or a bone-by-bone approach were compared. While using inspection by individual bones, estimates derived from all bones (30-boneGP) or uniquely from the pre-mature bones (GPpmb) were also compared. Finally, intra-individual mean differences were tested after calculating SAs using the mean or the median values from examined bone-specific SAs to obtain an individual SA estimate. It was hypothesized that agreement rates would be different when observers follow a wholeGP or bone-by-bone approaches.

## Methods

### Ethics and procedures

This cross-sectional, descriptive study was approved by the *Ethics Committee* for *Sports Sciences* in the *University of Coimbra* (Reference CE/FCDEF-UC/00122014). All data were collected in the *Porto Sports Medicine Center* as part of the medical exams for registration in the *Portuguese Soccer Federation* (Law 204/2006; act 11/2012). An institutional agreement was signed between the *University of Coimbra* and the *Portuguese Institute of Sports* [IPDJ/FCDEF.UC/2017–01]. Parents or legal guardians were informed about the aims, testing protocols, risks and provided informed consent. Participants were also informed about the nature of the research and that they were allowed to withdraw from the study at any time.

A standardized radiograph of the left hand-wrist was obtained by an experienced technician. SA was assessed using the GP method, which is often called the atlas method [13]. It is an inspectional protocol that was developed from a study of children from high socio-economic families in Ohio (Cleveland, USA). The method involves the matching of a specific radiograph of an observed participant to the closest plate from the collection of illustrations (photographs) representing a sequence of biological (skeletal) maturation. The estimate of SA refers to the CA of the child from the *Brush Foundation Study* whose plate was classified as the closest to the one under-examination. Thus, if the radiograph of a 13-year-old female soccer player matches the standard plate of the atlas obtained from a 11-year-old girl, the SA of the participant is 11 years. Each film was rated twice by an observer (OB1) who had completed a 45-h post-graduation course including the anatomy of hand and wrist, the biological basis of skeletal tissue, and the sequence of changes for each of the 30 bones assessed by the GP method. In parallel, measurements were also completed by a trained examiner (BO2) who had experience of conducting over 2000 assessments over the previous 3 years using the GP method in addition to other protocols (e.g. Tanner-Whitehouse; Fels). Examiners did not know the CA of the participants prior to applying the GP method.

### Participants

The sample for this study were 100 Portuguese female soccer players aged 12.0–16.7 years. To be included in the study participants were required to have played competitive soccer for at least 2 years in a club affiliated to the *Portuguese Soccer Federation*. Exclusion criteria were: (i) ≥17 years of age; (ii) any traumatic bone injury in the hand and left wrist that causes radiopaque lines or areas; (iii) previous/current exposure to medicines (e.g. steroids, growth hormone) that affects growth acceleration.

### Determination of SA

Each radiograph film was evaluated using three alternative protocols: inspection of the whole plate with the closest atlas photograph retained as the SA of the participant (wholeGP); 30 bones were individually examined and included in calculations of the SA (30-boneGP); 30 bones were individually examined and calculations to obtain the SA of the participant were based on pre-mature bones only (GPpmb). For 30-boneGP and GPpmb, SA was calculated using the mean (M) or, in alternative, the median (Md). Consequently, the following scores were produced: 30-boneGP-M, 30-boneGP-Md, GPpmb-M, GPpmb-Md.

### Analyses

Intra-observer reproducibility rates for each of the 30 bones were reported for each participant. Afterwards, inter-observer agreement was calculated for each bone and analyzed by maturity status (that is, agreement between two observers was examined separately for pre-mature and mature bones). The error (OB1 minus OB2) was calculated for each individual bone. Mean differences of the SAs rated by OB1 and OB2 were calculated and magnitudes of the differences interpreted as follows [16]: < 0.20 (trivial), 0.20 to 0.59 (small), 0.60 to 1.19 (moderate), 1.20 to 1.99 (large), 2.0 to 3.9 (very large), and > 4.0 (nearly perfect). SAs produced by OB1 and OB2 were plotted using scatter diagrams. Overlapping variance among examiners was determined using Pearson correlation coefficients which were interpreted as follows [16]: trivial (r < 0.1), small (0.1 < r < 0.3), moderate (0.3 < r < 0.5), large (0.5 < r < 0.7), very large (0.7 < r < 0.9), and nearly perfect (r > 0.9). Intra-class correlation coefficients were calculated to examine the variance between measurements for each observer. All analyses were performed using SPSS version 20 (SPSS, Inc. IBM Company; NY, USA) and Graphpad Prism (version 5 for Windows, GraphPad Software, San Diego California USA, www.graphpad.com).

## Results

Tables 1 and 2 summarizes the intra-observer agreement rates, respectively for OB1 and OB2. The less experienced observer (OB1) agreement rates were < 90% (i.e. for radius: 82%; ulna: 86%; metacarpal V: 89%; proximal phalange II: 89%; proximal phalange IV: 89%). Of these 66.8% were positive (time-moment 1 minus time-moment 2) and 87.5% of the total number of errors were − 1 or + 1 plates of the atlas (two consecutive stages – see Table 1). Identical analysis was performed by the more experienced observer (OB2) and results summarized in Table 2. When 30 bones were individually scored, intra-observer agreement rate was < 90% uniquely for proximal phalange I (88%) and 99% of the errors emerged from variation among two consecutive stages with a trend for lower SA values in the second examination (discrepancies − 1 stage: 61.1%; discrepancies + 1 stage: 23.3%). In other words, increasing practice, particularly in OB1, tended to produce slightly lower SA scores.

**Table 1:** Intra-observer error (observer 1) on SAs estimates among female adolescent soccer players (*n* = 100)

	Time-moment 1 (TM1)	Time-moment 2 (TM2)	Agreement		Disagreement						
			n	%	n	magnitude of discrepancies^a^					
						-3	−2	−1	+ 1	+ 2	+ 3
Radius	15.6 ± 1.2	15.5 ± 1.3	82	82%	18			5	12	1	
Ulna	16.1 ± 1.1	16.1 ± 1.1	86	86%	14			8	6		
Capitate	12.9 ± 0.3	12.9 ± 0.4	93	93%	7			4	3		
Hamate	12.8 ± 0.6	13.0 ± 0.2	92	92%	8	3	1	4			
Triquetral	12.8 ± 0.6	12.9 ± 0.5	90	90%	10	1	3	2	4		
Pisiform	–	–	100	100%							
Lunate	12.9 ± 0.4	12.9 ± 0.4	96	96%	4			1	2	1	
Scaphoid	12.9 ± 0.4	12.8 ± 0.5	91	91%	9			1	6	2	
Trapezium	12.8 ± 0.6	12.8 ± 0.6	90	90%	10	1	1		1	7	
Trapezoid	12.9 ± 0.4	12.9 ± 0.4	95	95%	5	1		1	2	1	
Adductor Sesamoid	–	–	100	100%							
Metacarpal I	14.9 ± 0.5	14.9 ± 0.5	94	94%	6			1	5		
Metacarpal II	14.7 ± 0.6	14.6 ± 0.6	91	91%	9			5	4		
Metacarpal III	14.7 ± 0.6	14.7 ± 0.6	92	92%	8			2	6		
Metacarpal IV	14.8 ± 0.5	14.7 ± 0.6	90	90%	10				10		
Metacarpal V	14.7 ± 0.5	14.7 ± 0.6	89	89%	11			3	6	2	
Proximal phalange I	14.4 ± 0.6	14.4 ± 0.6	91	91%	9		1	4	4		
Proximal phalange II	14.8 ± 0.5	14.7 ± 0.6	89	89%	11			1	10		
Proximal phalange III	14.8 ± 0.5	14.7 ± 0.6	91	91%	9				9		
Proximal phalange IV	14.8 ± 0.5	14.8 ± 0.6	89	89%	11		1	1	9		
Proximal phalange V	14.7 ± 0.6	14.8 ± 0.6	90	90%	10			8	2		
Medial phalange II	14.9 ± 0.4	14.8 ± 0.6	90	90%	10			1	8	1	
Medial phalange III	14.9 ± 0.5	14.8 ± 0.6	91	91%	9			1	7	1	
Medial phalange IV	14.9 ± 0.5	14.8 ± 0.5	91	91%	9			1	8		
Medial phalange V	14.8 ± 0.5	14.8 ± 0.6	92	92%	8			4	4		
Distal phalange I	14.3 ± 0.6	14.3 ± 0.6	97	97%	3			2	1		
Distal phalange II	14.3 ± 0.6	14.3 ± 0.6	95	95%	5			2	3		
Distal phalange III	14.3 ± 0.6	14.3 ± 0.6	96	96%	4			1	3		
Distal phalange IV	14.3 ± 0.6	14.3 ± 0.6	96	96%	4			1	3		
Distal phalange V	14.3 ± 0.5	14.3 ± 0.6	99	99%	1				1		
Total disagreements			2768		232	6	7	64	139	16	0
%			92.3%		7.7%	2.6%	3.1%	27.6%	59.9%	6.9%	0.0

^a^signs refer to TM1 minus TM2 and numbers corresponds to plates from the collection of photographs representing the sequence of skeletal maturation in atlas

**Table 2:** Intra-observer error (observer 2) on SAs estimates among female adolescent soccer players (*n* = 100)

	Time-moment 1 (TM1)	Time-moment 2 (TM2)	Agreement		Disagreement						
			n	%	n	magnitude of discrepancies^a^					
						−3	−2	−1	+ 1	+ 2	+ 3
Radius	15.6 ± 1.2	15.5 ± 1.2	90	90%	10			1	9		
Ulna	16.0 ± 1.1	16.0 ± 1.1	94	94%	6				6		
Capitate	12.9 ± 0.3	13.0 ± 1.2	96	96%	4			4			
Hamate	12.9 ± 0.5	13.0 ± 0.4	96	96%	4			4			
Triquetral	13.0 ± 0.2	13.0 ± 0.1	96	96%	4			4			
Pisiform	–	–	100	100%							
Lunate	12.9 ± 0.4	13.0 ± 0.2	91	91%	9			9			
Scaphoid	12.7 ± 0.6	12.8 ± 0.5	93	93%	7			7			
Trapezium	12.8 ± 0.6	12.8 ± 0.5	96	96%	4		1	3			
Trapezoid	12.9 ± 0.3	13.0 ± 0.1	96	96%	4			4			
Adductor Sesamoid	–	–	100	100%							
Metacarpal I	14.9 ± 0.4	14.9 ± 0.4	92	92%	8			1	7		
Metacarpal II	14.7 ± 0.6	14.6 ± 0.4	91	91%	9			2	7		
Metacarpal III	14.6 ± 0.6	14.6 ± 0.6	95	95%	5			3	2		
Metacarpal IV	14.6 ± 0.6	14.6 ± 0.6	96	96%	4			4			
Metacarpal V	14.6 ± 0.7	14.6 ± 0.6	94	94%	6			3	3		
Proximal phalange I	14.4 ± 0.7	14.4 ± 0.6	88	88%	12			7	5		
Proximal phalange II	14.8 ± 0.6	14.8 ± 0.6	98	98%	2			1	1		
Proximal phalange III	14.7 ± 0.7	14.8 ± 0.6	98	98%	2			2			
Proximal phalange IV	14.7 ± 0.7	14.8 ± 0.6	96	96%	4			4			
Proximal phalange V	14.7 ± 0.6	14.8 ± 0.6	96	96%	4			4			
Medial phalange II	14.8 ± 0.6	14.8 ± 0.6	98	98%	2				2		
Medial phalange III	14.8 ± 0.6	14.7 ± 0.6	96	96%	4				4		
Medial phalange IV	14.8 ± 0.5	14.8 ± 0.5	94	94%	6			3	3		
Medial phalange V	14.8 ± 0.5	14.8 ± 0.5	98	98%	2			1	1		
Distal phalange I	14.3 ± 0.5	14.3 ± 0.6	94	94%	6			6			
Distal phalange II	14.3 ± 0.5	14.3 ± 0.5	100	100%							
Distal phalange III	14.3 ± 0.5	14.3 ± 0.5	93	93%	7			4	3		
Distal phalange IV	14.3 ± 0.5	14.3 ± 0.6	96	96%	4			4			
Distal phalange V	14.3 ± 0.5	14.3 ± 0.6	95	95%	5			3	2		
Total disagreements			2856		144		1	88	55		
%			95.2%		4.8%		1%	61.1%	23.3%		

^a^signs refer to TM1 minus TM2 and numbers corresponds to plates from the collection of photographs representing the sequence of skeletal maturation in atlas

Inter-observer agreement rates are summarized in Table 3 for each bone and by maturity status (pre-mature or mature). The two observers had 100% agreement when scoring mature bones. The number of participants who had pre-mature bones were greater for radius (*n* = 70), ulna (*n* = 52) and distal phalanges I-V (*n* = 65). The agreement rates among the two observers were lower for the pre-mature carpals (capitate, hamate, triquetral, lunate, scaphoid, trapezium, trapezoid). Excluding the pisiform and adductor sesamoid, disagreement between observers for the 28 bones was 7.9% (221 occasions) of the observations. Table 4 showed that 80.3% of disagreements were − 1 stage and + 1 stage, 17.1% were − 2 stages and + 2 stages and 2.5% were − 3 stages and + 3 stages. In general, when SAs were not identical, the less experienced observer (OB1) tended to score higher SAs (69.2%). For the total sample, mean SAs obtained from OB1 and OB2 attained identical values with mean differences classified as trivial, except for triquetral (d = 0.450; small), scaphoid (d = 0.390; small), metacarpal (d = 0.364; small) and medial phalange IV (d = 0.201; small).

**Table 3:** Inter-observer agreement rates on the SAs according to skeletal classification as not mature or mature and for the total number of examinations among female adolescent soccer players (*n* = 100)

Bones	Age at mature state^1^	Not mature^a^				Mature^a^				Total			
		n	D (n)	A		n	D (n)	A		n	D (n)	A	
				n	%			n	%			n	%
Radius	17	70	8	62	89%	30	0	30	100%	100	8	92	92%
Ulna	17	52	3	49	94%	48	0	48	100%	100	3	97	97%
Capitate	13	5	2	3	60%	95	2	93	99%	100	4	96	96%
Hamate	13	5	3	2	40%	95	6	89	94%	100	9	91	91%
Triquetral	13	4	4	0	0%	96	6	90	94%	100	10	90	90%
Pisiform	a)									100	0	100	100%
Lunate	13	11	7	4	36%	89	0	89	100%	100	7	93	93%
Scaphoid	13	22	19	3	14%	78	0	78	100%	100	19	81	81%
Trapezium	13	17	15	2	10%	83	1	82	99%	100	16	84	84%
Trapezoid	13	5	4	1	20%	95	2	93	98%	100	6	94	94%
Adductor Sesamoid	b)									100	0	100	100%
Metacarpal I	15	4	1	3	75%	96	0	96	100%	100	1	99	99%
Metacarpal II	15	26	4	22	85%	74	3	71	96%	100	7	93	93%
Metacarpal III	15	30	8	22	73%	70	0	70	100%	100	8	92	92%
Metacarpal IV	15	30	10	20	67%	70	0	70	100%	100	10	90	90%
Metacarpal V	15	32	13	19	59%	68	0	68	100%	100	13	87	87%
Proximal phalange I	15	53	4	49	92%	47	5	42	89%	100	9	91	91%
Proximal phalange II	15	14	4	10	71%	86	0	86	100%	100	4	96	96%
Proximal phalange III	15	16	9	7	44%	84	0	84	100%	100	9	91	91%
Proximal phalange IV	15	17	6	11	65%	83	0	83	100%	100	6	94	94%
Proximal phalange V	15	17	3	14	82%	83	7	76	92%	100	10	90	90%
Medial phalange II	15	16	8	8	50%	84	0	84	100%	100	8	92	92%
Medial phalange III	15	18	9	9	50%	82	0	82	100%	100	9	91	91%
Medial phalange IV	15	15	5	10	67%	85	1	84	99%	100	6	94	94%
Medial phalange V	15	15	11	4	73%	85	6	79	93%	100	17	83	83%
Distal phalange I	15	65	4	61	94%	35	0	35	100%	100	4	96	96%
Distal phalange II	15	65	4	61	94%	35	2	33	94%	100	6	94	94%
Distal phalange III	15	65	4	61	94%	35	1	34	97%	100	5	95	95%
Distal phalange IV	15	65	4	61	94%	35	1	34	97%	100	5	95	95%
Distal phalange V	15	65	2	63	97%	35	0	35	100%	100	2	98	98%
Total disagreement. n		819	178	641		1981	43	1938		3000	221	2779	
%					78.3%				97.8%				92.6%

^a^skeletal maturity status by observer 2; D (disagreement between observer 1 and observer 2); A (agreement between observer 1 and observer 2)

^1^Mature state defined as detailed in Radiograph Atlas of Skeletal Development of the Hand and Wrist

**Table 4:** Descriptive (mean ± standard deviation) and comparison between observers for bone SAs among female adolescent soccer players (*n* = 100)

	Observer 1 (OB1)	Observer 2 (OB2)	Comparisons		Disagreement						
					n	magnitude discrepancies^a^					
			d	(qualitative)		−3	−2	−1	+ 1	+ 2	+ 3
Radius	15.6 ± 1.2	15.6 ± 1.2	0.001	(trivial)	8			1	6	1	
Ulna	16.1 ± 1.1	16.0 ± 1.1	0.090	(trivial)	6				5	1	
Capitate	12.9 ± 0.3	12.9 ± 0.3	0.001	(trivial)	5			3	2		
Hamate	12.8 ± 0.6	12.9 ± 0.5	0.180	(trivial)	10	3	1	3	2		1
Triquetral	12.8 ± 0.6	13.0 ± 0.2	0.450	(small)	11		6	3	2		
Pisiform	–	–									
Lunate	12.9 ± 0.4	12.9 ± 0.4	0.001	(trivial)	7			1	5	1	
Scaphoid	12.9 ± 0.4	12.7 ± 0.6	0.390	(small)	20			2	14	4	
Trapezium	12.8 ± 0.6	12.8 ± 0.6	0.001	(trivial)	18	1	2	1	7	7	
Trapezoid	12.9 ± 0.4	12.9 ± 0.3	0.001	(trivial)	6	1	1	1	2	1	
Adductor Sesamoid	–	–									
Metacarpal I	14.9 ± 0.5	14.9 ± 0.4	0.001	(trivial)	2		1	1			
Metacarpal II	14.7 ± 0.6	14.7 ± 0.6	0.001	(trivial)	7		7				
Metacarpal III	14.7 ± 0.6	14.6 ± 0.6	0.168	(trivial)	8				8		
Metacarpal IV	14.8 ± 0.5	14.6 ± 0.6	0.364	(small)	13				12	1	
Metacarpal V	14.7 ± 0.5	14.6 ± 0.7	0.165	(trivial)	13				12	1	
Proximal phalange I	14.4 ± 0.6	14.4 ± 0.7	0.001	(trivial)	9			5	4		
Proximal phalange II	14.8 ± 0.5	14.8 ± 0.6	0.001	(trivial)	4				4		
Proximal phalange III	14.8 ± 0.5	14.7 ± 0.7	0.165	(trivial)	9				9		
Proximal phalange IV	14.8 ± 0.5	14.7 ± 0.7	0.165	(trivial)	9				9		
Proximal phalange V	14.7 ± 0.6	14.7 ± 0.6	0.001	(trivial)	11			7	4		
Medial phalange II	14.9 ± 0.4	14.8 ± 0.6	0.197	(trivial)	8				7	1	
Medial phalange III	14.9 ± 0.5	14.8 ± 0.6	0.182	(trivial)	10				9	1	
Medial phalange IV	14.9 ± 0.5	14.8 ± 0.5	0.201	(small)	6			1	4	1	
Medial phalange V	14.8 ± 0.5	14.8 ± 0.5	0.001	(trivial)	10			6	4		
Distal phalange I	14.3 ± 0.6	14.3 ± 0.5	0.001	(trivial)	5			3	2		
Distal phalange II	14.3 ± 0.6	14.3 ± 0.5	0.001	(trivial)	6		1	3	2		
Distal phalange III	14.3 ± 0.6	14.3 ± 0.5	0.001	(trivial)	5		1	2	2		
Distal phalange IV	14.3 ± 0.6	14.3 ± 0.5	0.001	(trivial)	5			3	2		
Distal phalange V	14.3 ± 0.5	14.3 ± 0.5	0.001	(trivial)	3			1	2		
Diagreement. n					234	5	20	47	141	20	1
%						2.1%	8.5%	20.1%	60.2%	8.6%	0.4%

^a^signs refer to OB1 minus OB2 and numbers corresponds to plates from the collection of photographs representing the sequence of skeletal maturation in atlas

**Fig. 1:**
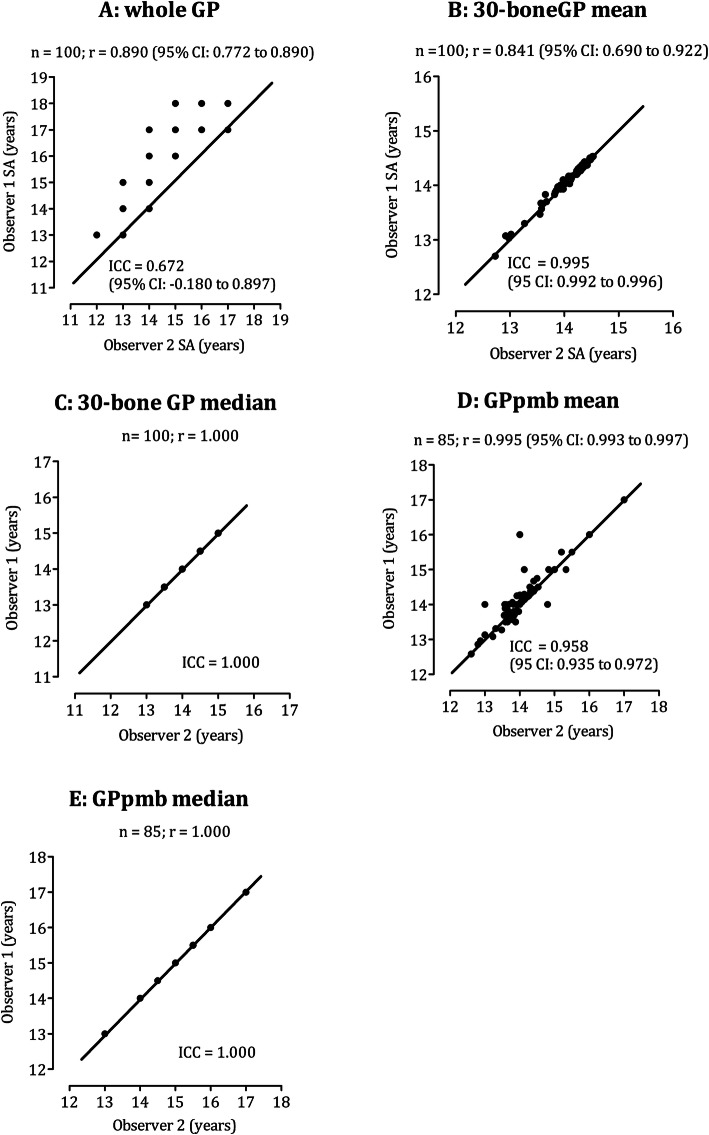
Scatterplot of the SA estimated by Observer 1 (y-axis) and Observer 2 (x-axis) in the whole inspection (**a**), a bone-by-bone approach using the mean (**b**) and, alternatively, the median (**c**) to calculate individual SA from all bone-specific SAs; and uniquely considering SAs of the pre-mature bones (**d** using the mean; **e** using the median)

Mean SAs calculated by the two observers are presented in Table 5. The mean SAs derived by the overall inspection (wholeGP) was 16.83 ± 1.30 years and 15.38 ± 1.22 years, respectively for OB1 and OB2 (d = 0.79; moderate mean differences). The bone-by-bone approaches attenuated differences between observers. When using the median, differences between observers resulted in no significant values and were considered as trivial (d = 0.10, for the pre-mature bones only; d = 0.05 for all 30 bones). Finally, independent from the observer, 30-boneGP using the median resulted in higher SAs compared to using the mean: + 0.36 years for OB1; + 0.38 years for OB2. When analyses used pre-mature bones only, mean and median values did not differ (Fig. 1d and e). The correlation coefficient between observers ranged from 0.841 (95% CI: 0.690 to 0.922) to 1.00 with the narrowest overlapping variance occurring when the exams followed bone-by-bone approach and calculations included both pre-mature and mature bones using the mean (30-boneGP-M).

**Table 5:** Descriptive statistics (mean ± standard deviation) of skeletal age estimates obtained from two observers and mean differences among female adolescent soccer players (*n* = 100)

Criteria			n	Observer 1	Observer 2	Mean difference		Magnitude effect	
Reading	processing	calculations							
						value	(95% CI)	d	(qualitative)
Overall	GPwhole		100	16.83 ± 1.30	15.38 ± 1.22	1.45	(1.30 to 1.59)	0.79	(moderate)
Bone-by-bone	30-boneGP	M	100	14.20 ± 0.36	14.18 ± 0.37	0.01	(0.09 to 0.30)	0.05	(trivial)
		Md	100	14.56 ± 0.61	14.56 ± 0.61	0.00	(0.00 to 0.00)		
	GPpmb	M	85#	14.24 ± 0.77	14.17 ± 0.74	0.07	(0.01 to 0.14)	0.10	(trivial)
		Md	85#	14.24 ± 0.76	14.16 ± 0.74	0.00	(0.00 to 0.00)		

*GPwhole* 30-bone assessment; *30-boneGP* All bones examined, *GPpmb* Only pre mature bones examined, *M* Mean, *Md* Median, *% 95 CI* 95% confidence intervals, *df* Degrees of freedom; # 15 participants presented all bones as mature; The paired t-test cannot be computed because the standard error of the difference between means is 0

## Discussion

Although Greulich-Pyle method has been often used to estimate SA from hand-wrist radiographs, little attention has been given to the impact of adopting different methodological approaches. The current study examined the reproducibility and agreement between two observers who assessed SAs of 100 female adolescent soccer players using the GP protocol. Disagreement between observers mostly occurred on carpal bones. Intra-observer agreement rates were acceptable, although the reproducibility was slightly lower for the less experienced. When differences existed, lower SAs were more likely to be derived in the second time-measurement. Finally, comparison between observers noted that the more experienced observer tended to produce slightly lower SA scores.

A previous study [17] of North-American children aged 4–15 years noted a lack of intra-observer agreement among carpal bones. It is plausible that agreement rates were associated with age, particularly before round bones (i.e. carpals) reach the mature state [18]. Carpals are more complex to rate compared to long bones [19], whose examinations concentrate on the centers of ossification and fusion of the epiphyseo-diaphysial. The examination of the carpal bones includes the inspection of the shape and radiopaque lines or zones which may help explain the poorer inter-observer agreement rates in the present study. Although mean differences between examiners in bone-specific SAs tended to be trivial or small, the number of disagreements (> 10%), were particularly apparent in the scaphoid and trapezium, metacarpals IV-V, and proximal phalange V. In general, the less experienced observer overestimated the ratings when compared to the experienced observer. Thus, less experienced examiners may need to adopt a conservative decision (i.e. when unsure match the radiograph to the younger of the two standard plates in the atlas). The literature includes considerable discussion about the exclusion of the carpal bones when a hand-wrist radiograph is assessed to obtain an estimate of SA [18–20].

The atlas method involves assigning a SA to each of the 30 bones of the hand-wrist [13]. The literature is not consistent regarding the appropriate utilization of the protocol. Consequently, GP SA is often assessed on the basis of an overall approach (that is, matching a film ignoring potential variation among bones [20, 21]. The current study examined alternative approaches such as only including 30 bones or pre-mature bones. The exclusion of mature bones is common in the literature [22, 23]. For example, Todd [24] recommended retention of the most advanced bones when calculating SA. In the present study, within each observer, the SA using the mean did not show fluctuations when considering the mature bones or not. Among Australian females, differences between GP SA mean from all bones and GP SA mean excluding the carpals were 0.02 and 0.05 for 12- and 13-year-old groups, respectively [22]. In the present study, the inter-individual variance was substantially reduced when the calculations were based on the mean and included mature bones (standard deviation was 0.36–0.37 years, depending of the observer). The largest standard deviation was seen in the overall (wholeGP) approach (standard deviation: 1.22–1.30 years). Recently, the median has been recommended as an alternative to the mean [1] to obtain the final SA from bone-specific SAs: “*The SA of the standard plate is the assigned SA of the bone in question. The process is repeated for all bones that are visible in the hand-wrist x-ray and the child’s SA is the median of the skeletal ages of each individually rated bone”* (pages 279–280).

There are a few limitations to note. The present study only included adolescent females, many of whom has mature carpals. Future studies need to consider younger samples and males. However, it should be noted that sport tends to focus on the middle and later adolescent years [2, 3, 6, 9], particularly in team sports such as soccer where competitive and organized participation tends to start after 11 years. Future research should focus on whether the impact of observer and methodological approach differs via child maturity status (i.e. early, average or late maturing).

## Conclusions

In summary, the GP method showed acceptable reproducibility and agreement between observers, suggesting that 45-h of training (rating 100 radiographs) is adequate. Where an observer is less experienced, he/she should be encouraged to select the younger age of two standards when the decision is not obvious. The BbB approach has a greater inter-observer agreement compared to the overall approach. Observers should organize their readings following a particular bone, instead of completing the scores by participant. Finally, the estimate of individual SA for each youth participant should use either the mean or the median of the pre-mature bones.

## Supplementary information

**Additional file 1.**

## Data Availability

The database supporting the conclusions of this article is available from the corresponding author on reasonable request.

## Abbreviations

CA: Chronological age
SA: Skeletal age
BA: Bone age
GP: Greulich-Pyle
d: Cohen’s d value
OB1: Observer 1
OB2: Observer 2
BbB: Bone-by-bone
wholeGP: Overall approach
30-boneGP: Bone-by-bone considering the 30-bones
GPpmb: Bone-by-bone considering the pre-mature bones
M: Mean
Md: Median
